# Electrocaloric effect in cubic Hubbard nanoclusters

**DOI:** 10.1038/s41598-018-23443-x

**Published:** 2018-03-23

**Authors:** Karol Szałowski, Tadeusz Balcerzak

**Affiliations:** Department of Solid State Physics, Faculty of Physics and Applied Informatics, University of Łódź, ulica Pomorska 149/153, PL90-236 Łódź, Poland

## Abstract

In the paper a computational study of the electrocaloric effect is presented for a cubic nanocluster consisting of 8 sites. The system of interest is described by means of an extended Hubbard model in external electric field at half filling of the energy levels. The thermodynamic description is obtained within canonical ensemble formalism on the basis of exact numerical diagonalization of the system Hamiltonian. In particular, the entropy and the specific heat are determined as a function of temperature and external electric field. The electrocaloric effect is described quantitatively by isothermal entropy change. The behaviour of this quantity is thoroughly analysed as a function of extended Hubbard model parameters, temperature and electric field variation magnitude. The existence of direct and inverse electrocaloric effect is predicted for some range of model parameters. A high sensitivity to Hubbard model parameters is shown, what paves the way towards controlling and tuning the effect. A non-linear, quadratic dependence of isothermal entropy change on electric field variation magnitude is demonstrated. The potential for applications of electrocaloric effect in strongly correlated nanoclusters is shown.

## Introduction

The dependence of the entropy in solids on such thermodynamic parameters as external fields or pressure gives rise to a variety of caloric effects^1–3^. Such phenomena are interesting not only because they provide some insight into the physics of various systems by probing the coupling between the system and the environment, but mainly due to high potential for applications in cooling. The most frequently studied phenomenon of this type is the magnetocaloric effect, emerging due to dependence of entropy on the magnetic field^4,5^. The less commonly explored phenomenon is the electrocaloric effect (ECE), which consists in the influence of the external electric field on the entropy of solid^6,7^. Such effect is recently suggested to possess remarkable advantages when applied in energy-conserving cooling systems^8,9^. Therefore, a search for novel materials exhibiting ECE with optimized parameters is well motivated^10,11^. In addition, the techniques necessary to measure the ECE-related quantities are undergoing constant development^12,13^. Simultaneous efforts concern mastering the theoretical description and modelling of ECE^14–24^.

The known electrocaloric materials include ferroelectrics like a prototypical one, barium titanate^25,26^, but also relaxor ferroelectrics^27–31^ (including polymers^32^), antiferroelectrics^33,34^, nanoparticles^15^ or ceramics^35^. However, it appears interesting to extend the studies of electrocaloric materials to new class of systems. This leads us to interest in strongly correlated materials, the physics of which can be essentially captured with the help of the canonical model called in such context - the Hubbard model^36^. Constituting a complicated many-body problem, the Hubbard model allows the exact solutions only for a range of low-dimensional systems. In particular, zero-dimensional clusters belong to that class, since for a finite and limited number of the lattice sites exact diagonalization approach can be effectively used to characterize the thermodynamics of the system. In that context numerous theoretical works can be mentioned^37–50^. Presenting a detailed studies of various thermodynamic properties, however, none of these studies was focused on electrocaloric quantities. Moreover, only a few included an external electric field within Hubbard model Hamiltonian, mainly focusing on the description of electric-field controlled magnetism in graphene-based materials^51,52^. The situation is slightly different in the case of the magnetocaloric effect, which has been a subject of some theoretical studies for the exactly solvable model spin systems. Such examples like zero-dimensional systems, in a form of nanoclusters^53–57^, should be mentioned in this context.

It should be stated that the study of the Hubbard model in the external electric field for the bulk material would be beyond the purpose of this paper. Instead, we are interested in a situation which is characteristic for small clusters, when most of the atoms are situated on the surface and they experience a direct influence of the external field, whereas the screening effects are negligible. The model cube, with the atoms situated in the corners, is an ideal candidate for such studies, since in this case all the atoms are located on the surface of the cluster, and the strongest interaction with the external field can be expected.

**Figure 1:**
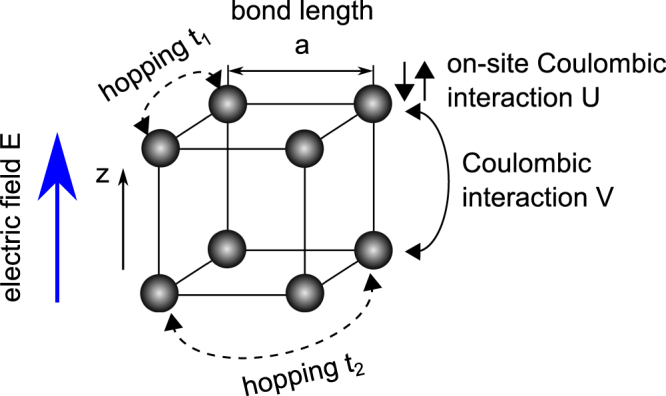
The schematic view of the system of interest - a cubic nanocluster consisting of 8 atoms, described within extended Hubbard model. The electron hoppings and coulombic interactions are shown schematically. The direction of the external electric field is marked.

As a consequence, the aim of our study is theoretical characterization of the electrocaloric effect in a model nanostructure, being a zero-dimensional nanocluster of cubic shape. It consists of *N* = 8 atoms, as shown schematically in Fig. 1. The bond length defining the distance between nearest neighbour atoms in the cube is *a*. The behaviour of the electrons in the system is described by means of the extended single-orbital Hubbard model. The presence of *n* = *N* = 8 electrons is assumed, so the half-filling condition for the electronic levels is satisfied. The electrons occupy only a single band. The cube is placed in external electric field *E* directed along one of the bonds - parallel to *z* direction in the scheme (Fig. 1). The values of *z* = 0 and *z* = *a* correspond to the lower and upper side of the cube, respectively.

The extended Hubbard model for the system in electric field has the following Hamiltonian:

1 $$\begin{array}{rcl} {\mathcal H}  & = & -{t}_{1}\sum _{\langle i,j\rangle ,\sigma }({c}_{i,\sigma }^{\dagger }{c}_{j,\sigma }+h\mathrm{.}c\mathrm{.})-{t}_{2}\sum _{\langle \langle i,j\rangle \rangle ,\sigma }({c}_{i,\sigma }^{\dagger }{c}_{j,\sigma }+h\mathrm{.}c\mathrm{.})\\  &  & +\,U\sum _{i}{n}_{i,\uparrow }{n}_{i,\downarrow }+V\sum _{\langle i,j\rangle ,\sigma ,\sigma ^{\prime} }{n}_{i,\sigma }{n}_{j,\sigma ^{\prime} }+\,\,eE\sum _{i,\sigma }{n}_{i,\sigma }{z}_{i}\mathrm{.}\end{array}$$

The index $i=\mathrm{1,}\ldots ,N$ labels the sites of the nanocluster, while 〈*i*, *j*〉 and 〈〈*i*, *j*〉〉 denote the pairs of nearest neighbours and second nearest neighbours, respectively. The spin variable is labelled with *σ* = ↑,↓. The operator ${c}_{i,\sigma }^{\dagger }\,\,\,({c}_{i,\sigma })$ creates (annihilates) an electron with spin *σ* at site *i*, whereas the operator of the number of electrons is ${n}_{i,\sigma }={c}_{i,\sigma }^{\dagger }{c}_{i,\sigma }$.

The fundamental Hubbard model is parametrized only by the energy of electronic hopping which takes place between nearest neighbours (hopping integral *t*_1_) and by the energy of on-site coulombic interactions between electrons *U*. However, the extended Hubbard model used here contains additional parameters. They are: the hopping integral for electronic hopping between the second nearest neighbours *t*_2_, as well as the energy of coulombic repulsion for nearest-neighbour electrons *V*. All the mentioned quantities are schematically marked in Fig. 1.

The uniform constant electric field *E* is introduced by its potential term $eE{z}_{i}/{t}_{1}$, in which *z*_*i*_ denotes the coordinate of the site *i* along *z* direction (see Fig. 1), while *e* is the elementary charge. The dimensionless potential is expressed in *t*_1_ units. For the dimensionless unit, *eEa*/*t*_1_ = 1, where *a* is the lattice constant, the approximate value of the electric field *E* can be estimated as 1 V/Å. In that context, it can be mentioned that the studies of nanoclusters for this range of fields are present in the literature^58^.

## Results

### Entropy and specific heat

**Figure 2:**
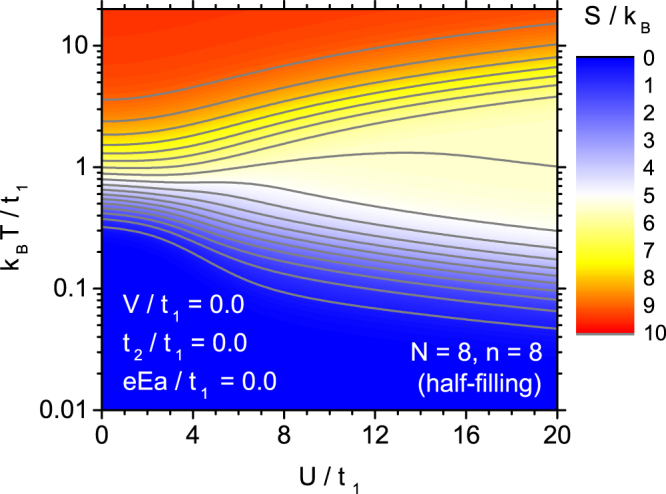
The contour plot of entropy for Hubbard model without extensions, as a function of the normalized temperature and normalized coulombic on-site parameter, in the absence of the external electric field.

The most fundamental thermodynamic quantity from the point of view of description of any caloric effect is the entropy and its dependence on the temperature. The entropy is defined as, $S=-{(\partial G/\partial T)}_{E}$ where *G* is the Gibbs free energy. Therefore, we commence the presentation of the numerical data with the plot of normalized entropy as a function of the temperature and the on-site coulombic interaction parameter, which is shown in Fig. 2. This contour plot presents the results obtained for the case of the Hubbard model without extensions and in the absence of the electric field. Please note the logarithmic temperature scale used to emphasize the low-temperature behaviour. The limiting value of reduced entropy at *T* = 0 is equal to 0, while in the high temperature limit it is equal to $\mathrm{ln}\,{N}_{s}=\,\mathrm{ln}\,12870\simeq 9.463$. It is visible that for low on-site coulombic parameter the entropy increase between the low value (close to zero) and high value (close to the limiting one) takes place in a rather limited interval of moderate temperatures. The effect of increasing *U* is seen as the widening of this interval of temperatures, so that the isolines of constant entropy shift towards lower temperatures for low entropy and show the opposite behaviour for high entropy as the on-site coulombic parameter increases.

**Figure 3:**
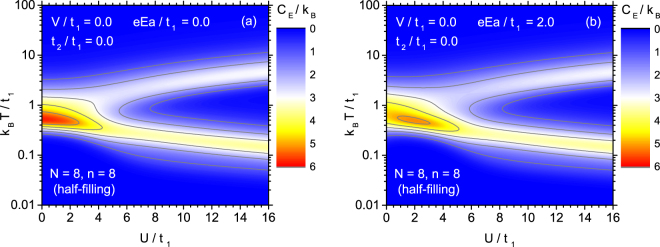
The contour plot of the specific heat at constant electric field for Hubbard model without extensions, as a function of the normalized temperature and normalized coulombic on-site parameter. Two cases of the normalized electric field are considered: $eaE/{t}_{1}=0.0$ (**a**) and $eaE/{t}_{1}=2.0$ (**b**).

In order to emphasize some more subtle features of the temperature dependence of entropy, it is instructive to refer to the specific heat *C*_*E*_ defined by Eq. (7), i.e., ${C}_{E}=T{(\partial S/\partial T)}_{E}={k}_{{\rm{B}}}{\beta }^{2}(\langle { {\mathcal H} }^{2}\rangle -{\langle  {\mathcal H} \rangle }^{2})$, as the derivative is more sensitive in this case. Moreover, *C*_*E*_ is an interesting quantity itself as a thermal response function. The effect of the on-site coulombic parameter on the temperature dependence of the specific heat can be followed in Fig. 3, again in the form of the contour plot. The case of Fig. 3(a) corresponds to the absence of the electric field. For low *U* a pronounced single peak of specific heat is observed at moderate temperatures, which is correlated with the interval of the fastest entropy change. However, if *U* is increased, the peak tends to lose its magnitude and becomes shifted towards lower temperatures. For sufficiently strong on-site coulombic energy a second maximum emerges, which becomes shifted to higher temperatures under the influence of increasing *U*. This structure with two peaks exhibiting the opposite tendencies to shift when the on-site coulombic parameter changes, explains the behaviour of entropy discussed in the context of Fig. 2, when the temperature interval in which entropy varies most significantly expands with increase of *U*.

A similar behaviour of the specific heat can be followed in Fig. 3(b), which illustrates the case of the non-zero electric field, $eEa/{t}_{1}=2$. The emerging double-peaked structure of the temperature dependence of *C*_*E*_ is conserved in the presence of *E*. However, the range of low *U* is mostly affected, because the magnitude of specific heat maximum is reduced there. Moreover, the maximum of *C*_*E*_ as a function of *U*, occurring at moderate temperatures, is shifted to non-zero value of *U*-parameter. The high-*U* behaviour of the specific heat is rather weakly influenced by the presence of the electric field.

**Figure 4:**
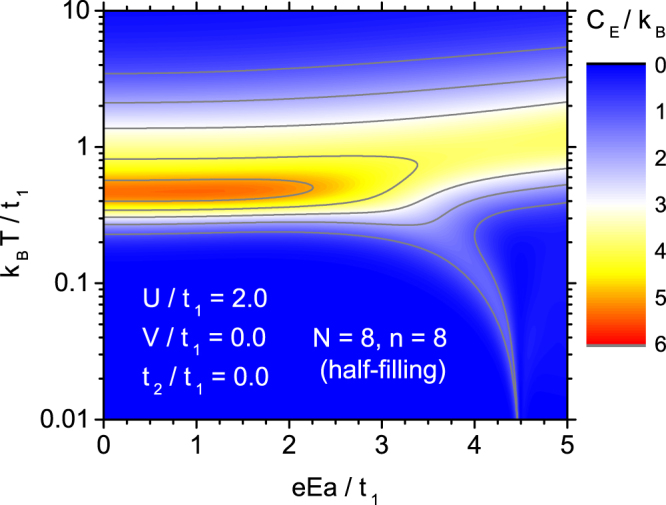
The contour plot of the specific heat *C*_*E*_, for Hubbard model without extensions and constant coulombic *U*-parameter, *U*/*t*_1_ = 2, vs. electric field *E* and normalized temperature *T*.

It is instructive to follow the temperature variability of specific heat as a function of the electric field, for fixed coulombic on-site parameter. Such an analysis can be performed on the basis of Fig. 4, which presents the case of unmodified Hubbard model with weak on-site parameter, *U*/*t*_1_ = 2. For these parameters and low fields *E*, a single peak of specific heat occurs at intermediate temperatures. Application of the electric field in general tends to reduce the peak magnitude, but this effect is not very pronounced for weak fields. For stronger *E* the maximum of *C*_*E*_ is shifted somehow towards higher temperatures and an additional branch being a weak second maximum emerges for a narrow interval of electric fields. For the case of stronger on-site coulombic interactions (not shown in figures), the influence of the electric field is even less pronounced, with a general tendency to reduce the magnitude of specific heat peaks. The second, weak maximum, which is seen in the low-temperature region in the vicinity of the field $eEa/{t}_{1}\approx 4.5$ does not exist in the limit ${k}_{{\rm{B}}}T/{t}_{1}\to 0$ but it finishes close to ${k}_{{\rm{B}}}T/{t}_{1}\approx {10}^{-3}$ for the set of Hamiltonian parameters in Fig. 4. It has been checked that in the limit ${k}_{{\rm{B}}}T/{t}_{1}\to 0$ the specific heat *C*_*E*_ always tends to zero value, in accordance with the third law of thermodynamics. This particular behaviour of the specific heat can be interrelated with the ground-state properties of the system. In Electronic Supplementary Material we provide Fig. S1, which presents a dependence of the energy gap between the ground state and the first excited state on the electric field (with an inset showing an analogous dependence of the individual energy states of interest). At the point $eEa/{t}_{1}\approx 4.5$ the gap closes and for stronger electric fields it remains rather small, moreover, the ground state acquires three-fold degeneracy.

In order to verify the influence of the energy of coulombic interactions between electrons localized at nearest-neighbour sites *V* on the specific heat, we include Fig. S2 in Electronic Supplementary Material, which is a contour plot of *C*_*E*_ as a function of the temperature and normalized repulsion *V* between nearest neighbours, in the absence of electric field, for *U*/*t*_1_ = 2.

### Isothermal entropy change

**Figure 5:**
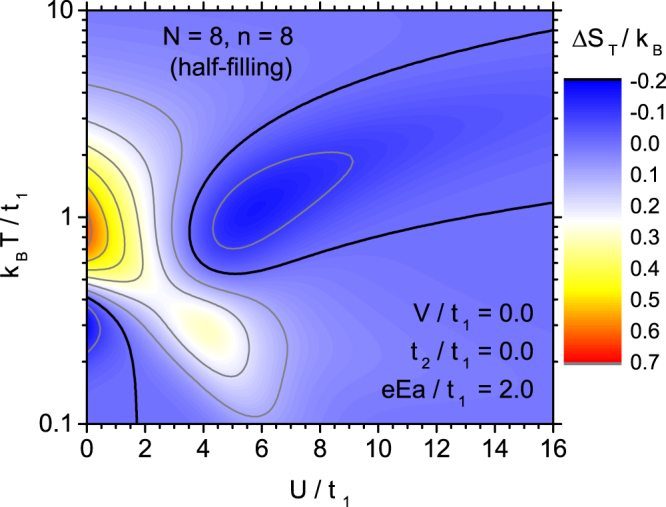
The contour plot of isothermal entropy change for Hubbard model without extensions, as a function of the normalized temperature and normalized coulombic on-site parameter. The bold contour line separates the range of normal ECE (Δ*S*_*T*_ > 0) and inverse ECE (Δ*S*_*T*_ < 0).

Let us commence the characterization of electrocaloric properties from the systematic analysis of the effect of coulombic interaction *U* on the isothermal entropy change, ${\rm{\Delta }}{S}_{T}=S(T,E=0)-S(T,E)$, when the electric field is varied between fixed non-zero value *E* and zero value. The isothermal entropy change is usually considered as a quantity characterizing the vertical size of Ericsson cycle^9^, when this cycle is presented in (*T*,*S*) coordinates, and therefore is very important for the description of electrocaloric effect, being also used to define refrigerant capacity^7,9^. This quantity is also interesting from the point of view of its dependence on the electric field strength *E*. Let us only mention that alternatively, the derivative $({\rm{\partial }}S/{\rm{\partial }}E{)}_{T}$ could also be studied. Both quantities are related by ${\rm{\Delta }}{S}_{T}={\int }_{E}^{0}{(\frac{\partial S(T,E^{\prime} )}{\partial E^{\prime} })}_{T}\,dE^{\prime}$. It can be mentioned that the entropy change for finite electric field variation magnitude can be directly measured for example with differential scanning calorimetry technique^7,12,59^. First, an unmodified Hubbard model will be considered, i.e. with *t*_2_ = 0 and *V* = 0. In order to visualise the effect we present a contour plot in Fig. 5, showing the isothermal entropy change in the temperature-coulombic interaction energy plane (please note the logarithmic temperature scale). In general, it can be noticed that both ranges of direct ECE (with Δ*S*_*T*_ > 0) and inverse ECE (corresponding to Δ*S*_*T*_ < 0) are present in our system of interest. One inverse ECE range emerges at low temperatures for rather weak coulombic interactions. Another, much more extensive range is present at intermediate temperatures and it appears only when *U*/*t*_1_ exceeds a certain critical value. If the coulombic interaction rises further, the range shifts toward higher temperatures, but the magnitude of inverse ECE achieves a maximum around $U/{t}_{1}\simeq 6$ and then falls down. The rest of the phase diagram is filled with the range of direct ECE, which is always present at high enough or low enough temperature (excluding the range of low values of *U*). However, at a certain range of coulombic interaction energy, only the direct ECE is found at arbitrary temperature. The direct ECE magnitude is the largest at intermediate temperature and for weak or zero *U*/*t*_1_. Another maximum (smaller) is present at low temperatures and *U*/*t*_1_ close to 4. What is important, the extremal magnitude of direct ECE is about 3 times larger than the corresponding magnitude of inverse ECE.

As it is seen from the above analysis, the electrocaloric effect is rather sensitive to the energy of the coulombic interactions, which are capable of modifying severely its magnitude as well as of switching between the direct and inverse ECE at given temperature, and also of moving the position of its extrema.

Further control of the behaviour of ECE can be expected owing to extension of the Hubbard model by including the electronic hopping to second nearest neighbours *t*_2_ as well as the coulombic interactions between nearest-neighbour electrons *V*. Both parameters are capable of shaping the thermal dependence of the isothermal entropy change.

**Figure 6:**
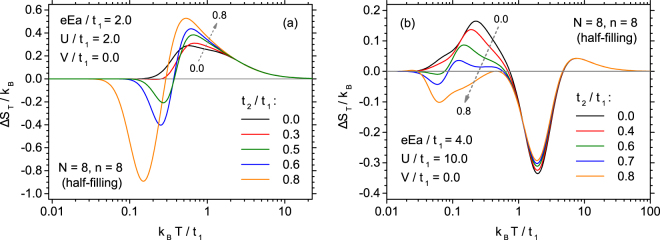
The effect of the increasing normalized hopping between second nearest neighbours on the thermal dependence of isothermal entropy change, for extended Hubbard model with low on-site coulombic interactions parameter *U*/*t*_1_ = 2 (**a**) and for high on-site coulombic interactions parameter *U*/*t*_1_ = 10 (**b**).

First the effect of inclusion of *t*_2_ will be discussed. In Fig. 6(a) the evolution of the thermal dependence of entropy change is shown for low value of on-site coulombic parameter *U*/*t*_1_ = 2. Under such conditions, only direct ECE is present for *t*_2_ = 0 (see Fig. 5). It is visible that by introducing *t*_2_ the extremal magnitude of direct ECE tends to increase. Moreover, in the low-temperature range, a region of inverse ECE builds up, with formation of a minimum on Δ*S*_*T*_ curve. This minimum shifts towards lower temperatures and increases in magnitude when *t*_2_ rises. Therefore, for *U*/*t*_1_ = 2 a profound influence of second nearest neighbours hopping on the character of ECE can be expected in the whole range of temperatures.

The situation is rather different for the case of stronger coulombic interactions, for instance, for *U*/*t*_1_ = 10, depicted in Fig. 6(b). In this case, increase in *t*_2_ barely affects the high-temperature minimum of ECE, just reducing slightly its magnitude without shifting the position. On the contrary, the low-temperature maximum corresponding to direct ECE gradually flattens and even a range of inverse ECE emerges for the lowest temperatures, forming a minimum, which further develops. This proves that for stronger coulombic interactions only the low-temperature ECE can be effectively controlled and tuned with *t*_2_.

**Figure 7:**
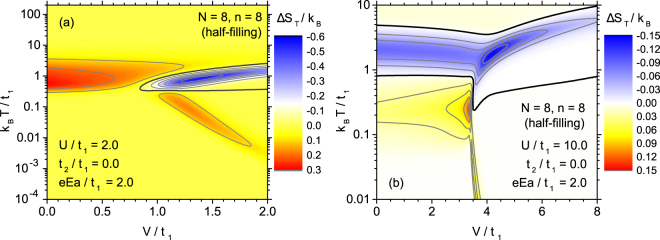
The effect of the increasing normalized repulsion *V* between nearest neighbours on the thermal dependence of isothermal entropy change for extended Hubbard model with low on-site coulombic interactions parameter *U*/*t*_1_ = 2 (**a**) and for high on-site coulombic interactions parameter *U*/*t*_1_ = 10 (**b**). The bold contour line separates the range of normal ECE (Δ*S*_*T*_ > 0) and inverse ECE (Δ*S*_*T*_ < 0).

Another parameter of the extended Hubbard model is the energy of coulombic interactions between electrons localized at nearest-neighbour sites *V*. The influence of this parameter on the form of ECE in the studied system, for low energy of coulombic on-site interactions *U*/*t*_1_ = 2 and *t*_2_ = 0 is visualized as a contour plot in Fig. 7(a). For Hubbard model without extensions we deal with only direct ECE. Under the influence of increasing *V* the maximum at intermediate temperatures gradually flattens and shifts somehow to higher temperatures. Then, for $V/{t}_{1}\simeq 1$ the different behaviour emerges - the range of direct ECE is divided into two ranges separated with inverse ECE with a minimum of Δ*S*_*T*_. When *V* grows further, this minimum develops, whereas the low temperature maximum of direct ECE moves to lower temperatures and the high-temperature maximum of ECE flattens. As a consequence, a profound effect of including *V* on the isothermal entropy change in ECE is found.

The case of stronger coulombic on-site interactions, for *U*/*t*_1_ = 10, appears more complex, as it is seen in Fig. 7(b). When weak *V* > 0 is present, both the low-temperature maximum of direct ECE and the high-temperature minimum of inverse ECE are not greatly influenced by *V.* However, above *V*/*t*_1_ = 3, the low-temperature range of direct ECE disappears completely, just after passing through a very pronounced peak. At the same time the high-temperature minimum of inverse ECE becomes shifted towards higher temperatures, what is accompanied with decrease in magnitude.

**Figure 8:**
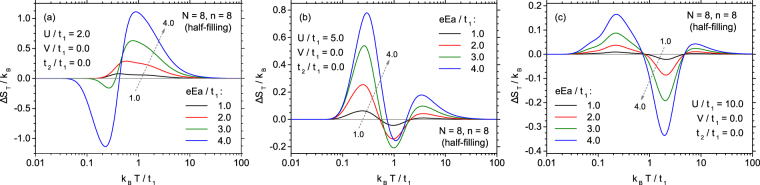
The dependence of the isothermal entropy change on the normalized temperature, for various normalized magnitudes of the external electric field change. *U*/*t*_1_ = 2 (**a**), *U*/*t*_1_ = 5 (**b**), and *U*/*t*_1_ = 10 (**c**).

A crucial feature of the electrocaloric effect is the behaviour of its quantitative measures as a function of the magnitude of electric field change. Such an analysis can be performed on the basis of Fig. 8, for three cases of on-site coulombic interactions, *U*/*t*_1_ = 2 (Fig. 8(a)), 5 (Fig. 8(b)) and 10 (Fig. 8(c)). In the case of low coulombic interactions, *U*/*t*_1_ = 2.0 (Fig. 8(a)), it is visible that for low electric field only a broad range of direct ECE is present. When the field increases, this maximum develops and the temperature at which entropy change reaches the extremal magnitude rises slowly. For even stronger field amplitude, the low-temperature range of inverse ECE builds up, being very sensitive to *E*. It can be, therefore, seen, that for low coulombic on-site interactions energy the amplitude of electric field not only influences the magnitude of ECE but also changes qualitatively the shape of its temperature dependence. On the contrary, for stronger coulombic on-site interactions (shown in Fig. 8(b) and (c)), only the magnitude of Δ*S*_*T*_ is varied when the electric field amplitude changes, while the general shape of the dependence of Δ*S*_*T*_ on *T* is conserved, and no switching between direct and inverse ECE takes place. In that cases a pronounced nonlinearity in the dependence of the entropy change on the electric field can be observed, as Δ*S*_*T*_ rises faster than linearly with *E*.

In order to supplement the physical picture, also the thermal variability of the differential ECE measure, ${(\partial S/\partial E)}_{T}$ for various electric fields can be traced, as in Fig. S3 in Electronic Supplementary Material. In general, the curves bear significant similarity to the corresponding ones for the quantity Δ*S*_*T*_ (Fig. 8). Moreover, the contour plot of $({\rm{\partial }}S/{\rm{\partial }}E{)}_{T}$ as a function of temperature and electric field is presented as Fig. S4.

**Figure 9:**
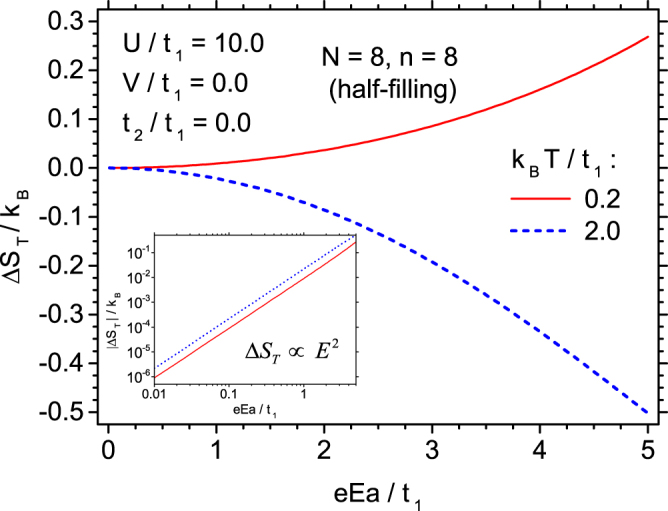
Dependence of the isothermal entropy change on the normalized electric field amplitude at two fixed temperatures, corresponding to the extremal direct and inverse ECE for *U*/*t*_1_ = 10.0. In the inset the absolute value of isothermal entropy change is plotted as a function of the normalized electric field amplitude in double logarithmic scale, to emphasize the quadratic dependence.

In order to get more detailed insight into the variability of Δ*S*_*T*_, let us plot this quantity as a function of the electric field amplitude for a fixed temperature. For this purpose we select $U/{t}_{1}=10.0$ and the temperatures corresponding approximately to the extrema of ECE (both direct one, at lower temperature, and inverse one at higher temperature). The dependence is presented in Fig. 8 (in main plot the linear scale is used) and the non-linear increase of the entropy change magnitude with the increase of the electric field can be clearly confirmed. The non-linear variability calls for plotting the data in double logarithmic scale, what is done in the inset to Fig. 9 (where the absolute value of Δ*S*_*T*_ is presented). In double logarithmic scale both dependences linearize and it can be extracted from the inset that the dependence is quadratic, that is $|{\rm{\Delta }}{S}_{T}|\propto {E}^{2}$, for the studied ranges of parameters (close to the extrema). This is an important finding, since, for example, in the case of the usually considered magnetocaloric effect, the entropy change is linearly dependent on the magnetic field amplitude.

The above quadratic dependence means that the derivative $\partial S/\partial E$ is linear vs. *E* in the considered range of parameters. In turn, taking into account the Maxwell relation: $\partial S/\partial E=\partial P/\partial T$, where *P* is the electric polarization of the cluster, we see that *P* should be also a linear function of *E*. This fact proves that our system is in a range of linear response to the external force. For the discussion of the quadratic dependence of the ECE measure on electric field see also ref.^33^.

## Discussion

In the paper a theoretical study of the electrocaloric effect in a cubic nanocluster embedded in the electric field is presented. The system is modelled with the help of extended Hubbard model at half-filling, taking into account coulombic interactions between on-site and nearest-neighbour electrons, as well as electronic hopping between nearest- and next nearest neighbours. For the resulting Hamiltonian an exact numerical diagonalization was performed within canonical ensemble formalism, for fixed number of electrons. Such situation corresponds to chemically isolated system, where the chemical potential is constant and no exchange of electrons with the environment takes place. It should be mentioned here that for nanoscopic systems interacting chemically with their environment, for instance, when the cluster interacts with the conducting substrate, the grand canonical ensemble should be more appropriate. Both ensembles are applied in the studies of nanoscopic Hubbard systems and they are inequivalent^60,61^. When the statistical fluctuations of the number of electrons are taken into account within grand canonical ensemble, for small clusters, the discussed properties are additionally influenced. We might state that both ensembles correspond to manifestly different physical situations and their comparison would inspire further works. The thermodynamic description included in particular such quantities as the specific heat and entropy, which are the functions of the external electric field and temperature. In order to characterize the ECE, the isothermal entropy change was thoroughly studied as a function of temperature, Hubbard model parameters and electric field amplitude. Significant ranges of both direct and inverse ECE were identified. Moreover, significant sensitivity of the isothermal entropy change to Hubbard model parameters was shown. This enables the design and control of the electrocaloric properties of the nanoclusters.

It should be mentioned that having obtained the entropy as a function of *T* and *E*, the change of temperature (Δ*T*)_*S*_ can be studied, when the field *E* is switched on (off) adiabatically, i.e., for $S=const\mathrm{.}$ The parameter (Δ*T*)_*S*_ has a meaning for the prediction of cooling (heating) effect in the system, when rapid changes of the external field take place. However, in this paper we concentrated rather on the comprehensive investigations of the (ΔS)_*T*_ and *C*_*E*_ quantities. We would like to emphasize that the knowledge of the isothermal entropy change allows the determination of the heat transfer to or from the system, which is the vital process during the electrocaloric refrigeration.

It should be particularly emphasized that the obtained results are exact, since they originate from the exact numerical diagonalization of the many-body extended Hubbard Hamiltonian for the system in question. Such an approach is computationally demanding, what limits its applicability to small systems, however, it provides a rigorous description of the thermodynamic properties, capturing all the many-body effects.

In this paper we concentrate on the rigorous solutions of the extended Hubbard model for small clusters when the electric field *E* is applied. In this model approach the influence of the electric field on the Hubbard Hamiltonian parameters was neglected, and these parameters are considered to be constant. This is the usual approach (e.g.^62,63^). However, it should be noted here that for the electric field with the potential changing markedly on the scale of lattice constant, the atomic states which give rise to the Hubbard model could be deformed in comparison with the states without field. The example of the study when the atomic 1*s* states were modified to follow the varying interatomic distance parameter was given, for example, in ref.^64^. As a result, this deformation under the action of the electric field will influence both the hopping integrals as well as the Coulomb interaction potentials. Therefore, one should be aware that for very strong electric fields, the Hubbard model with constant interaction parameters can serve only as an approximate description of the physical reality, being, on the other hand, a starting point for more sophisticated treatments of the problem.

The future developments may include, for example, the consideration of various cluster sizes and shapes, as well as larger structures (with the help of modified numerical approach to the Hubbard model). Another potentially fruitful direction involves the study of charge doping on the ECE in nanostructures. Moreover, another external field such as magnetic field could be incorporated to investigate multicaloric properties^65^. In addition, lattice-related degrees of freedom might be also taken into account to exploit possible coupling to lattice entropy.

## Methods

In order to obtain the full thermodynamic description of the system, an exact diagonalization method is used, allowing the treatment of the Hubbard terms in the Hamiltonian without any approximations^66^. The exact numerical results are obtained at the cost of considerable computational complexity: for the case of *N* = 8 lattice sites with *n* = 8 electrons (half-filling), the Hilbert space is spanned by ${N}_{s}=\frac{(2N)!}{n!(2N-n)!}=12870$ states. Therefore, the resulting Hamiltonian matrix for diagonalization is of the size $({N}_{s}\times {N}_{s})$. It should be mentioned that the number of dimensions of the Hilbert space for given *N* is maximized at half filling.

The model Hamiltonian ${\mathcal H}$ for the system in question is subject to exact numerical diagonalization^67^, yielding the eigenvalues *E*_*k*_ with $k=\mathrm{1,}\ldots ,{N}_{s}$ and the corresponding eigenvectors.

The exact thermodynamic description of our system is constructed within the canonical ensemble, with the external electric field taken into account, assuming fixed number of electrons *n* and the fixed temperature equal to *T*. Under such assumptions, the statistical sum is given by:

2 $${\mathscr{Z}}={\rm{Tr}}\,{e}^{-\beta  {\mathcal H} }=\sum _{k}{e}^{-\beta {E}_{k}},$$

where $$\beta =\mathrm{1/}({k}_{{\rm{B}}}T)$$.

The knowledge of the statistical sum allows the direct calculation of the Gibbs free energy:

3 $$G=-{k}_{{\rm{B}}}T\,\mathrm{ln}\,{\mathscr{Z}}\mathrm{.}$$

On the other hand, the enthalpy of the system is given as the thermal average of the Hamiltonian:

4 $$H=\langle  {\mathcal H} \rangle =\frac{1}{{\mathscr{Z}}}\sum _{k}{E}_{k}\,{e}^{-\beta {E}_{k}}\mathrm{.}$$

Let us put emphasis on the fact that the Hamiltonian given by Eq. (1) contains the potential energy term for the electrons in the electric field, therefore, its average $\langle  {\mathcal H} \rangle$ is not a sole internal energy but enthalpy instead. For the same reason the free energy determined directly from the statistical sum is the Gibbs free energy and not the Helmholtz one.

The knowledge of both quantities i.e., *G* and *H*, yields the entropy value, which is the most interesting quantity for us in the present study. Namely:

5 $$S=\frac{H-G}{T}\mathrm{.}$$

The entropy $S(T,E)$ is then given exactly for the system of interest, as a function of the temperature and external electric field, for the whole range of the extended Hubbard model parameters.

The knowledge of entropy allows, among other things, the study of the electrocaloric effect (ECE). Its crucial measure is the isothermal entropy change when the external electric field varies at constant temperature from the value *E* = 0 to *E* > 0, i.e.

6 $${\rm{\Delta }}{S}_{T}=S(T,E=0)-S(T,E)\mathrm{.}$$

If the value of Δ*S*_*T*_ is positive, so the electric field decreases the entropy of the system, the direct ECE occurs. If the opposite case takes place, it is a manifestation of inverse ECE.

Another quantity of interest may be the specific heat of the system for constant electric field ${C}_{E}=T{(\partial S/\partial T)}_{E}$, which can be conveniently calculated on the basis of fluctuation-dissipation theorem, namely:

7 $${C}_{E}={k}_{{\rm{B}}}{\beta }^{2}(\langle { {\mathcal H} }^{2}\rangle -{\langle  {\mathcal H} \rangle }^{2})\mathrm{.}$$

We note that for the diagonalized Hamiltonian the average $\langle { {\mathcal H} }^{2}\rangle$ can be calculated analogously like $\langle  {\mathcal H} \rangle$ in Eq. (4), namely:

8 $$\langle { {\mathcal H} }^{2}\rangle =\frac{1}{{\mathscr{Z}}}\sum _{k}{E}_{k}^{2}\,{e}^{-\beta {E}_{k}}\mathrm{.}$$

## Electronic supplementary material

Supplementary Material

## References

[1] Crossley S, Mathur ND, Moya X (2015). New developments in caloric materials for cooling applications. AIP Advances.

[2] Takeuchi I, Sandeman K (2015). Solid-state cooling with caloric materials. Physics Today.

[3] Mañosa L, Planes A, Acet M (2013). Advanced materials for solid-state refrigeration. Journal of Materials Chemistry A.

[4] Tishin, A. M. & Spichkin, Y. I. *The Magnetocaloric Effect and its Applications* (CRC Press, 2016).

[5] Franco V, Blázquez JS, Ingale B, Conde A (2012). The Magnetocaloric Effect and Magnetic Refrigeration Near Room Temperature: Materials and Models. Annual Review of Materials Research.

[6] Correia, T. & Zhang, Q. Electrocaloric Effect: An Introduction. In Correia, T. & Zhang, Q. (eds.) *Electrocaloric Materials* 1–15 (Springer Berlin Heidelberg, 2014).

[7] Kutnjak, Z., Rožič, B., Pirc, R. & Webster, J. G. Electrocaloric Effect: Theory, Measurements, and Applications. In *Wiley Encyclopedia of Electrical and Electronics Engineering* (John Wiley & Sons, Inc., 1999).

[8] Plaznik U (2015). Electrocaloric cooling: The importance of electric-energy recovery and heat regeneration. EPL.

[9] Ožbolt M, Kitanovski A, Tušek J, Poredoš A (2014). Electrocaloric refrigeration: Thermodynamics, state of the art and future perspectives. International Journal of Refrigeration.

[10] Valant M (2012). Electrocaloric materials for future solid-state refrigeration technologies. Progress in Materials Science.

[11] Scott JF (2011). Electrocaloric Materials. Annual Review of Materials Research.

[12] Liu Y, Scott JF, Dkhil B (2016). Direct and indirect measurements on electrocaloric effect: Recent developments and perspectives. Applied Physics Reviews.

[13] Kutnjak, Z. & Rožič, B. Indirect and Direct Measurements of the Electrocaloric Effect. In Correia, T. & Zhang, Q. (eds.) *Electrocaloric Materials* 147–182 (Springer Berlin Heidelberg, 2014).

[14] Shi Y (2017). A scaling law for distinct electrocaloric cooling performance in low-dimensional organic, relaxor and anti-ferroelectrics. Scientific Reports.

[15] Wang F (2017). Inhomogeneous electric-field–induced negative/positive electrocaloric effects in ferroelectric nanoparticles. EPL.

[16] Liu M, Wang J (2015). Giant electrocaloric effect in ferroelectric nanotubes near room temperature. Scientific Reports.

[17] Herchig R, Chang C-M, Mani BK, Ponomareva I (2015). Electrocaloric effect in ferroelectric nanowires from atomistic simulations. Scientific Reports.

[18] Ma Y-B, Albe K, Xu B-X (2015). Lattice-based Monte Carlo simulations of the electrocaloric effect in ferroelectrics and relaxor ferroelectrics. Physical Review B.

[19] Beckman SP, Wan LF, Barr JA, Nishimatsu T (2012). Effective Hamiltonian methods for predicting the electrocaloric behavior of BaTiO_3_3. Materials Letters.

[20] Rose MC, Cohen RE (2012). Giant Electrocaloric Effect Around *T*_*c*_. Physical Review Letters.

[21] Ponomareva I, Lisenkov S (2012). Bridging the Macroscopic and Atomistic Descriptions of the Electrocaloric Effect. Physical Review Letters.

[22] Zhang J, Heitmann AA, Alpay SP, G. AR (2011). Aspects of the Electrocaloric Behavior of Ferroelectric Thin Films: A Review of the Predictions of the Landau-Ginzburg Theory. Integrated Ferroelectrics.

[23] Lisenkov S, Ponomareva I (2009). Intrinsic electrocaloric effect in ferroelectric alloys from atomistic simulations. Physical Review B.

[24] Prosandeev S, Ponomareva I, Bellaiche L (2008). Electrocaloric effect in bulk and low-dimensional ferroelectrics from first principles. Physical Review B.

[25] Moya X (2013). Giant Electrocaloric Strength in Single-Crystal BaTiO_3_. Advanced Materials.

[26] Novak N, Pirc R, Kutnjak Z (2013). Impact of critical point on piezoelectric and electrocaloric response in barium titanate. Physical Review B.

[27] Jiang Z (2017). Electrocaloric effects in the lead-free Ba(Zr,Ti)O_3_ relaxor ferroelectric from atomistic simulations. Physical Review B.

[28] Lu, B. *et al*. Large Electrocaloric Effect in Relaxor Ferroelectric and Antiferroelectric Lanthanum Doped Lead Zirconate Titanate Ceramics. *Scientific Reports* **7**, 45335 (2017). 10.1038/srep45335 PMC5366905 28345655

[29] Suchaneck G, Gerlach G (2015). Electrocaloric cooling based on relaxor ferroelectrics. Phase Transitions.

[30] Pirc R, Kutnjak Z, Blinc R, Zhang QM (2011). Electrocaloric effect in relaxor ferroelectrics. Journal of Applied Physics.

[31] Dunne LJ, Valant M, Axelsson A-K, Manos G, Alford NM (2011). Statistical mechanical lattice model of the dual-peak electrocaloric effect in ferroelectric relaxors and the role of pressure. Journal of Physics D: Applied Physics.

[32] Li, X., Lu, S.-G., Qian, X., Lin, M. & Zhang, Q. M. Electrocaloric Polymers. In *Electrocaloric Materials* 107–124 (Springer, Berlin, Heidelberg, 2014).

[33] Lisenkov S, Mani BK, Glazkova E, Miller CW, Ponomareva I (2016). Scaling law for electrocaloric temperature change in antiferroelectrics. Scientific Reports.

[34] Pirc R, Rožič B, Koruza J, Malič B, Kutnjak Z (2014). Negative electrocaloric effect in antiferroelectric PbZrO_3_. EPL.

[35] Goupil FL (2016). Tuning the electrocaloric enhancement near the morphotropic phase boundary in lead-free ceramics. Scientific Reports.

[36] Tasaki H (1998). The Hubbard model - an introduction and selected rigorous results. Journal of Physics: Condensed Matter.

[37] Rycerz A (2017). Pairwise entanglement and the Mott transition for correlated electrons in nanochains. New Journal of Physics.

[38] Souza TXR, Macedo CA (2016). Ferromagnetic Ground States in Face-Centered Cubic Hubbard Clusters. PLOS ONE.

[39] Alfonsi J, Lanzani G, Meneghetti M (2010). Exact diagonalization of Hubbard models for the optical properties of single-wall carbon nanotubes. New Journal of Physics.

[40] Schumann R, Zwicker D (2010). The Hubbard model extended by nearest-neighbor Coulomb and exchange interaction on a cubic cluster – rigorous and exact results. Annalen der Physik.

[41] López-Urías F, Pastor GM (1999). Exact numerical study of the ground-state magnetic properties of clusters. Physical Review B.

[42] Pastor GM, Hirsch R, Mühlschlegel B (1996). Magnetism and structure of small clusters: An exact treatment of electron correlations. Physical Review B.

[43] Pastor GM, Hirsch R, Mühlschlegel B (1994). Electron correlations, magnetism, and structure of small clusters. Physical Review Letters.

[44] Callaway J, Chen DP, Kanhere DG, Li Q (1990). Small-cluster calculations for the simple and extended Hubbard models. Physical Review B.

[45] Parola A, Sorella S, Baroni S, Parrinello M, Tosatti E (1989). Static properties of the 2D Hubbard model on a 4 × 4 cluster. International Journal of Modern Physics B.

[46] Callaway J, Chen DP, Tang R (1987). Ground-state and thermodynamic properties of the Hubbard model applied to small clusters. Physical Review B.

[47] Callaway J, Chen DP, Zhang Y (1987). Hubbard model for a cubic cluster. Physical Review B.

[48] Falicov LM, Victora RH (1984). Exact solution of the Hubbard model for a four-center tetrahedral cluster. Physical Review B.

[49] Spałek J, Oleś AM, Chao KA (1979). Thermodynamic properties of a two-site Hubbard model with orbital degeneracy. Physica A.

[50] Oleś AM, Spałek J, Chao KA (1979). Thermodynamic properties of small extended Hubbard rings. Physica A.

[51] Luo K, Sheng W (2015). Bias voltage control of magnetic phase transitions in graphene nanojunctions. Nanotechnology.

[52] Szałowski K (2015). Graphene nanoflakes in external electric and magnetic in-plane fields. Journal of Magnetism and Magnetic Materials.

[53] Karl’ová K, Strečka J, Richter J (2017). Enhanced magnetocaloric effect in the proximity of magnetization steps and jumps of spin-1/2 XXZ Heisenberg regular polyhedra. Journal of Physics: Condensed Matter.

[54] Karl’ová K, Strečka J (2017). Magnetization Process and Magnetocaloric Effect of the Spin-1/2 XXZ Heisenberg Cuboctahedron. Journal of Low Temperature Physics.

[55] Strečka J, Karl’ová K, Madaras T (2015). Giant magnetocaloric effect, magnetization plateaux and jumps of the regular Ising polyhedra. Physica B.

[56] Žukovič M (2015). Thermodynamic and magnetocaloric properties of geometrically frustrated Ising nanoclusters. Journal of Magnetism and Magnetic Materials.

[57] Schnack J, Heesing C (2013). Application of the finite-temperature Lanczos method for the evaluation of magnetocaloric properties of large magnetic molecules. The European Physical Journal B.

[58] Zhao J, Yang J, Hou JG (2003). Theoretical study of small two-dimensional gold clusters. Physical Review B.

[59] Bai Y, Ding K, Zheng G-P, Shi S-Q, Qiao L (2012). Entropy-change measurement of electrocaloric effect of BaTiO_3_ single crystal. Phys. Status Solidi A.

[60] Fernando GW, Palandage K, Kocharian AN, Davenport JW (2009). Pairing in bipartite and nonbipartite repulsive Hubbard clusters: Octahedron. Physical Review B.

[61] Kocharian AN, Fernando GW, Palandage K, Davenport JW (2008). Coherent and incoherent pairing instabilities and spin-charge separation in bipartite and nonbipartite nanoclusters: Exact results. Physical Review B.

[62] Chowdhury J, Karmakar SN, Bhattacharyya B (2009). Effect of external electric field on the charge density waves in one-dimensional Hubbard superlattices. Journal of Physics: Condensed Matter.

[63] Dutta S, Pati SK (2008). External electric field mediated quantum phase transitions in one-dimensional charge-ordered insulators: a density matrix renormalization group study. Journal of Physics: Condensed Matter.

[64] Spałek J, Podsiadły R, Wójcik W, Rycerz A (2000). Optimization of single-particle basis for exactly soluble models of correlated electrons. Physical Review B.

[65] Ursic H (2016). A multicaloric material as a link between electrocaloric and magnetocaloric refrigeration. Scientific Reports.

[66] Weiϐe, A. & Fehske, H. Exact Diagonalization Techniques. In *Computational Many-Particle Physics* 529–544 (Springer, Berlin, Heidelberg, 2008).

[67] Wolfram, S. Wolfram Mathematica (Version 8.0.4). Wolfram Research, Inc., Champaign, Illinois, USA. www.wolfram.com/ (2010).

