# Supplementary material for: Electrocaloric effect in cubic Hubbard nanoclusters
Source: Sci Rep. 2018 Mar 23;8:5116. doi: 10.1038/s41598-018-23443-x (PMC5865123; doi:10.1038/s41598-018-23443-x)
Supplement: Supplementary file 1 — Supplementary Material [file 41598_2018_23443_MOESM1_ESM.pdf]

Electrocaloric effect in cubic Hubbard nanoclusters

ELECTRONIC SUPPLEMENTARY MATERIAL

Karol Szałowski[1,*] and Tadeusz Balcerzak[1]

[1]Department of Solid State Physics, Faculty of Physics and Applied Informatics, University of Łódź, ulica Pomorska 149/153, PL90-236 Łódź, Poland
[*]kszalowski@uni.lodz.pl

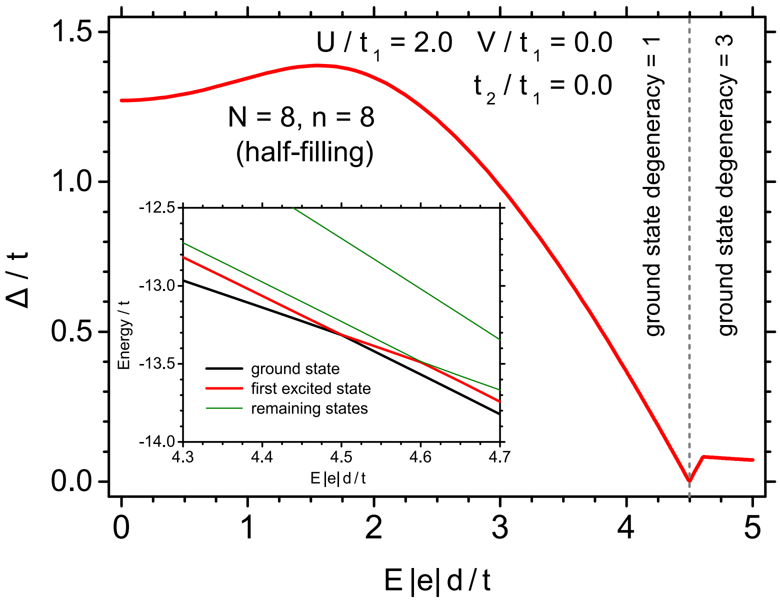

Figure S1. The energy gap between ground state and the first excited state for Hubbard model without extensions with $U/t_1 = 2$, as a function of the normalized electric field. The inset shows the lowest-lying energy states for the same model in a restricted range of normalized electric fields where the gap tends to close.

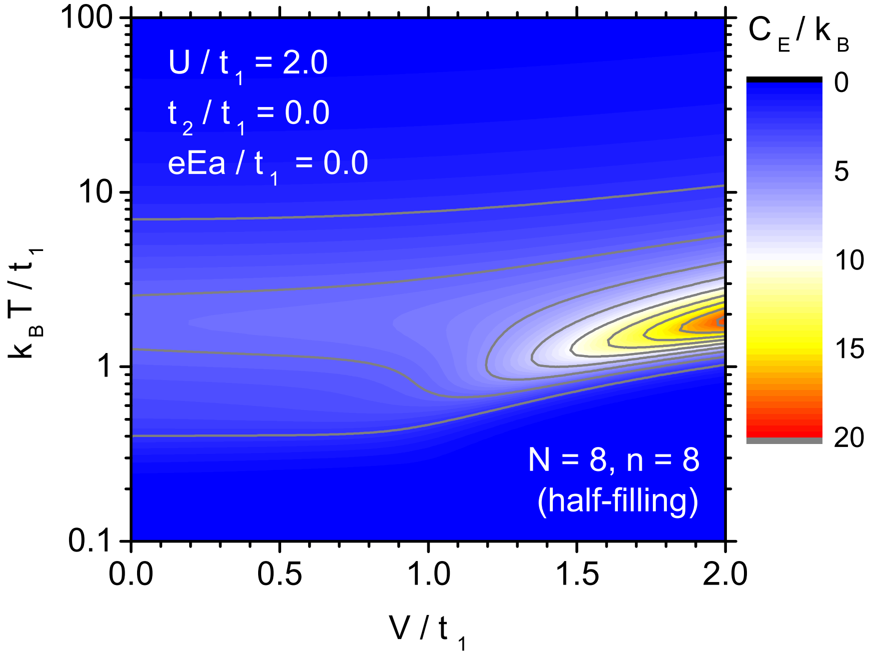

Figure S2. The contour plot of the specific heat C_E, for extended Hubbard model with $U/t_1 = 2$, vs. normalized repulsion V between nearest neighbours and normalized temperature T.

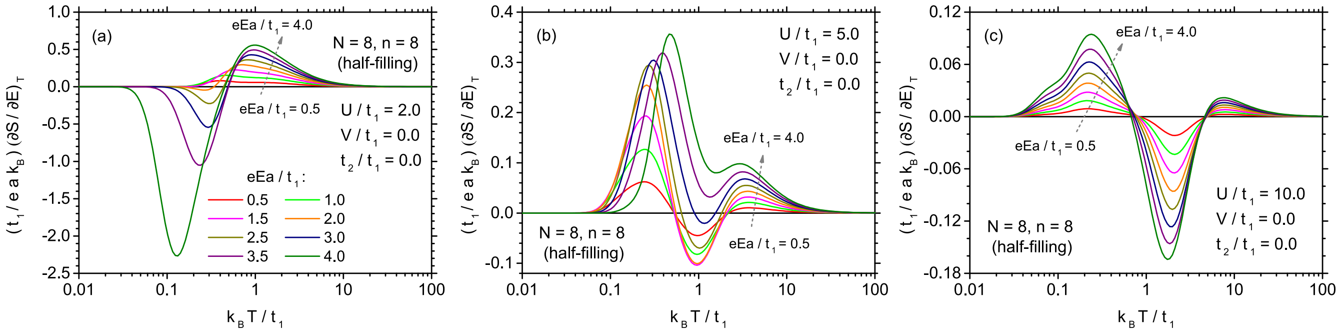

Figure S3. The dependence of the normalized derivative of entropy over the electric field for constant temperature, $(\partial S/\partial E)_T$, on the normalized temperature, for various normalized magnitudes of the external electric field, for Hubbard model without extensions. The on site coulombic interaction parameters are: $U/t_1 = 2$ (a), $U/t_1 = 5$ (b), and $U/t_1 = 10$ (c).

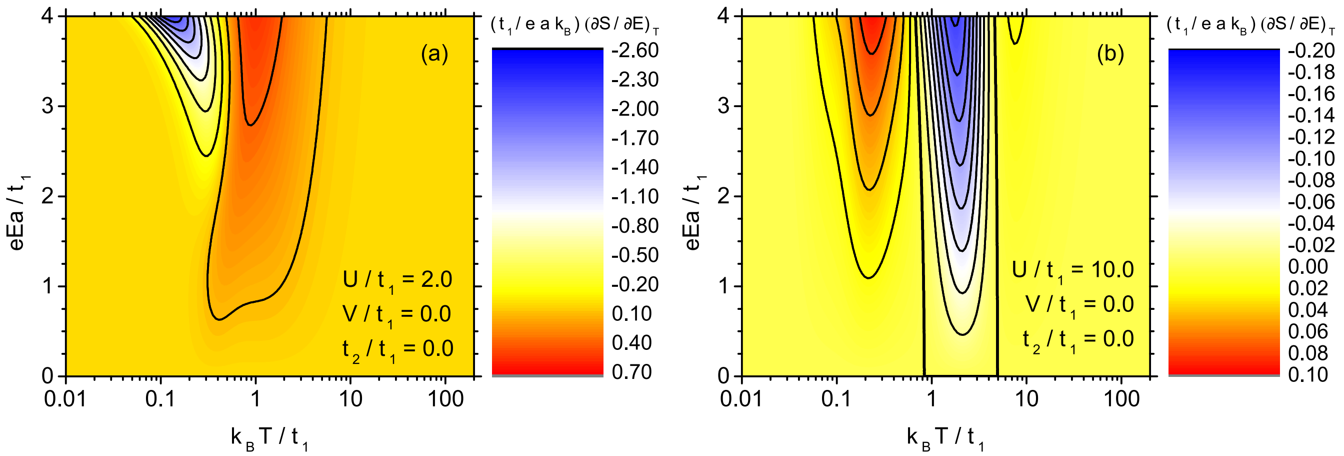

Figure S4. The contour plot of the normalized derivative of entropy over the electric field for constant temperature, $(\partial S/\partial E)_T$, for Hubbard model without extensions, as a function of the normalized temperature and normalized electric field. The on site coulombic interaction parameters are: (a) $U/t_1 = 2$, (b) $U/t_1 = 10$.
